# Experimental Investigations of a Precision Sensor for an Automatic Weapons Stabilizer System

**DOI:** 10.3390/s17010023

**Published:** 2016-12-24

**Authors:** Igor Korobiichuk

**Affiliations:** Industrial Research Institute for Automation and Measurements PIAP, Jerozolimskie 202, 02-486 Warsaw, Poland; kiv_Igor@list.ru; Tel.: +48-516-593-540

**Keywords:** accelerometer, weapons stabilizer system, mobile objects

## Abstract

This paper presents the results of experimental investigations of a precision sensor for an automatic weapons stabilizer system. It also describes the experimental equipment used and the structure of the developed sensor. A weapons stabilizer is designed for automatic guidance of an armament unit in the horizontal and vertical planes when firing at ground and air targets that are quickly maneuvering, and at lower speeds when firing anti-tank missiles, as well as the bypass of construction elements by the armament unit, and the automatic tracking of moving targets when interacting with a fire control system. The results of experimental investigations have shown that the error of the precision sensor developed on the basis of a piezoelectric element is 6 × 10^−10^ m/s^2^ under quasi-static conditions, and ~10^−5^ m/s^2^ for mobile use. This paper defines metrological and calibration properties of the developed sensor.

## 1. Introduction

Weapons stabilizer is an automatic control system that provides combat vehicles with weapon targeting and stabilization in the target fire direction during oscillation of a moving armoured vehicle (AV) [1]. To increase the effectiveness of fire during movement in all modern tanks and other combat vehicles, the main armament is stabilized by a special device referred to as a weapons stabilizer.

AV oscillations are random and never dampen in motion. The amplitude of angular oscillations and oscillation frequencies are quite high [2]. The accuracy of shooting is mostly influenced by AV oscillations in the longitudinal plane, changing the angle of gun elevation, and angular oscillations in the horizontal plane, changing the traverse [3]. This leads to a significant displacement of the aiming mark towards the target and does not allow the gunner to keep it on target even with the most advanced power drives. Lateral angular oscillations causing gun trunnion tilt have less impact, but increase with an extension of the firing range.

First of all, these causes increase vectoring errors by 10–30 times due to firing on the move, as compared with firing from a halt. Dispersion of projectiles increases by about 10–12 times [4]. Precision of fire on the move also decreases due to continuous changes in the target range. AV oscillations in motion significantly worsen firing conditions [5].

To increase the effectiveness of fire on the move, all modern combat vehicles are equipped with a special automatic device referred to as a weapons stabilizer system (WSS) [6]. Providing the accuracy improvement of measuring the mobile object’s acceleration, such as those used in a weapons stabilization system (WSS), is a great challenge [6]. WSS effectiveness is mostly dependent on the accuracy and performance of the sensitive stabilizer elements and accelerometers. The modern stabilization systems, using spring, string, quartz, magnetic, and gyroscopic accelerometers cannot provide the required speed of response and accuracy [7,8].

Therefore, the urgent scientific and technical challenge is to improve the accuracy and speed of response when measuring the acceleration values by experimental investigations of a piezoelectric sensor (PS) for the automatic weapons stabilization system [9,10,11]. The recent literature concerning this subject [12,13,14,15,16,17,18,19,20] contain no information on the analysis of experimental investigations of precision sensors of an automated weapons stabilizer system.

**Figure 1:**
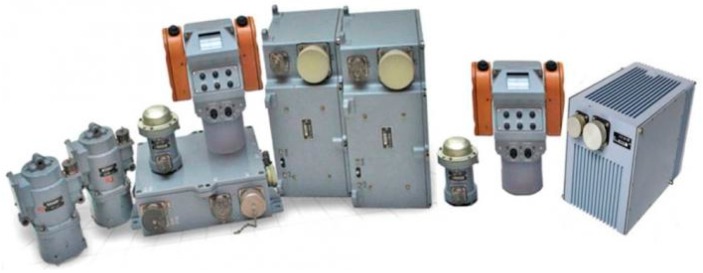
SVU-500 Weapon stabilising system (from left to right: SVU-500, SVU-500-01, SVU-500-3TS, SVU-500-4TS, SVU-500-4TS-01, SVU-500-10P, SVU-500-3TS-01).

The task given by the Ministry of Education and Science of Ukraine grant No. 0115U000210 was to improve the accuracy and error characteristics of the precision accelerometer sensor for WSS. The WSS under consideration was the SVU-500 (Figure 1), produced by the G. Petrovsky Kyiv Automatics Plant (Kyiv, Ukraine).

The WSS of this type are used in the fire control systems of the “Shkval”, on BTR 3E, on infantry fighting vehicles BMP IM (SVU-500-3TS), and in “Shturm” and “Parus” systems for APCs BTR1 3E and BMP 4 (SVU-500-4TS) [21].

The modernized version of SVU-500 is intended to be mounted on the AVs, such as the BMP-2 (BMP-2, BTR, BMP, BMD), to stabilize targeting and improve fire control during motion on land and on water. The existing WSS was using obsolete vibrating string accelerometers; therefore, a new approach was chosen, based on piezoelectric elements. The new sensor had to be compatible with existing WSS systems, which gave initial assumptions for the development process.

## 2. Measurement Stand

**Figure 2:**
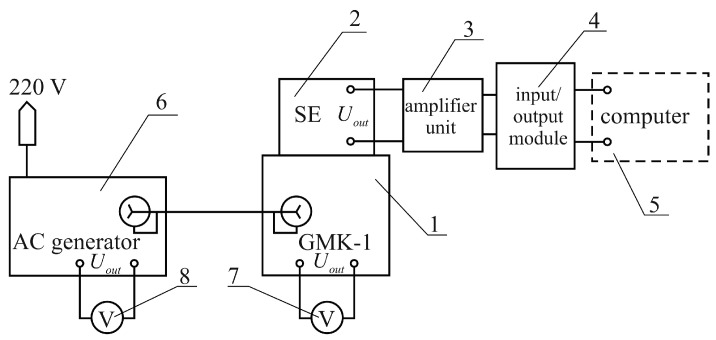
Schematic diagram of the test stand for the experimental investigation of piezoelectric SE: 1—GMK-1; 2—SE; 3—SE output signal amplifier unit; 4—input/output module; 5—PC; 6—AC generator; 7—voltmeter for logging generator voltage; and 8—voltmeter for logging induction transducer voltage.

**Figure 3:**
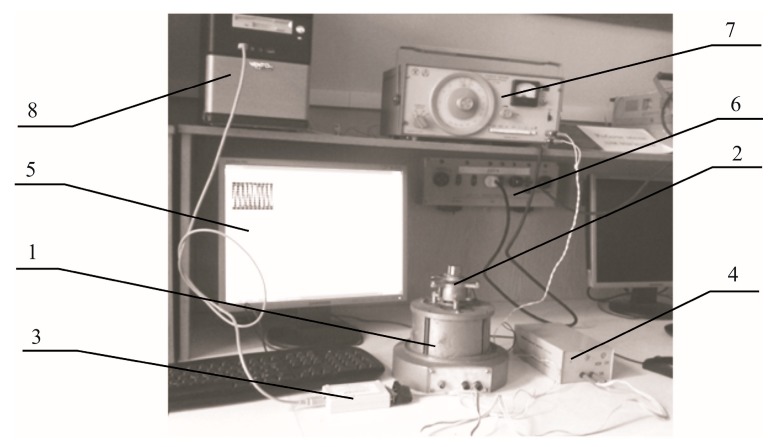
Appearance of the device for SE experimental investigations: 1—GMK-1; 2—SE; 3—SE output signal amplifier unit; 4—input/output module; 5—PC; 6—AC generator; 7—voltmeter for logging generator voltage; and 8—voltmeter for logging induction transducer voltage.

Experimental device was created for experimental investigations of the sensing element (SE). Its schematic diagram is shown in Figure 2, and its photo is presented in Figure 3. The test stand includes the following devices: a mechanical vibration generator GMK-1 (vibration table) with two on-board induction transducers converting electrical signals into mechanical displacement; an SE placed directly on the vibration table; an input/output module; an SE output signal amplifier unit; a personal computer (PC); an AC generator and voltmeters for logging voltage levels of the generator and of the induction transducers.

**Figure 4:**
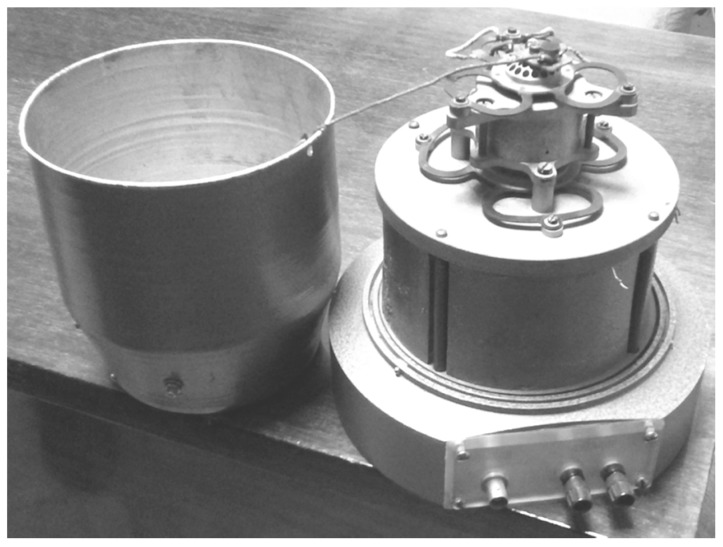
Mechanical vibration generator GMK-1.

**Figure 5:**
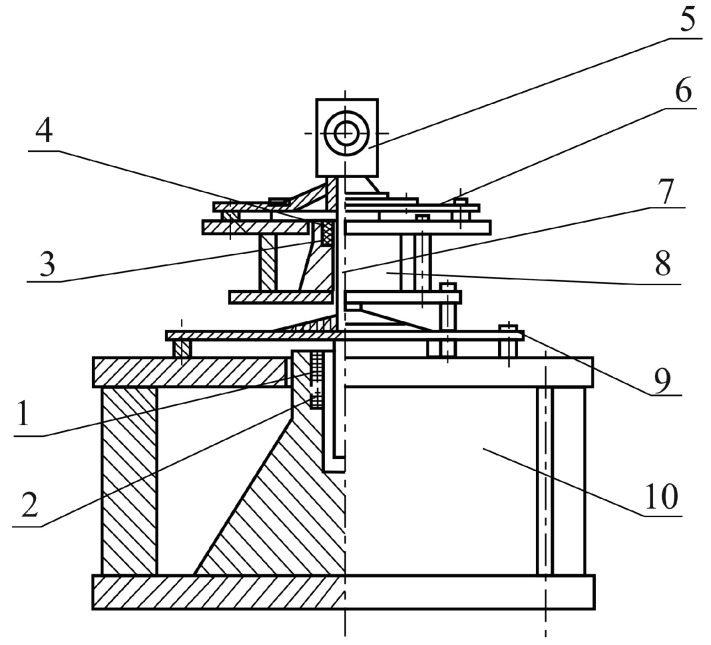
Mechanical vibration generator: 1, 3—generator windings; 2, 4—control winding; 5—working table with piezoelectric sensing element; 6, 9—flexible membranes; 7—rod; and 8, 10—magnetic cores.

The basis of the experimental test stand is the vibration table GMK-1 (Figure 4). It is a mechanical vibration generator, structurally designed as two magnetic cores (8, 10) (see Figure 5). These magnetic cores are tightly fastened and form a single structure of solenoid type. The rod (7) can move in the middle of a solenoid created by two magnetic cores (8, 10).

Driving force for moving the rod (7) is generated by induction transducers. Windings (1, 3) perform the excitation function and (2, 4) perform the control function.

Induction transducers shown in the diagram are designed to convert input electrical excitation signals into the output mechanical rod movement.

The core (7) with windings (1, 2) and (3, 4) is restrained by flexible supports in the form of special membranes (6, 9) which combine sufficient stiffness with great linear power curve values.

Fixation of the rod by membranes on both sides allows minimization of movement in directions that do not coincide with the longitudinal axis. This will provide the rod with only one degree of freedom in the required direction of the vertical axis. Therefore, if a current is passed through the generator winding, the power generated by the winding will result in a vertical movement of the rod.

Thus, the vibration table GMK-1, creating oscillatory rod acceleration, affects the working table (5), where the SE is found.

If current, varying in time sinusoidally, is passed through the generator winding (1) of the vibration table, the power generated by the winding will result in rod movement *h*, which is also sinusoidal:

(1) h=Hsinωt,

where *H*, *ω* = *2πf* are the amplitude and frequency of the oscillation movement of the rod, respectively.

Oscillating rod movement rate *h* is connected with values of rod oscillation velocity *h_c_* and acceleration *h_z_* affecting the SE by the following relations:

(2) $$h_{z}=-H\omega \sin\omega =H_{z}\sin\omega t,$$

(3) $$h_{c}=H\omega \cos\omega =H_{c}\cos\omega t.$$

Only amplitude values of the rod oscillation velocity and acceleration are measured during experimental investigations. As a result we obtain:

(4) $$H_{c}=Hn\omega ,$$

(5) $$H_{z}=-H_{c}\omega =Hn\omega^{2}.$$

Measurement transducer (2) has much smaller dimensions than the generator’s and is designed to measure acceleration amplitudes of the vibration table rod oscillation.

Amplitude of output voltage $U_{mt}$ of measurement transducer (2) winding is connected with the acceleration amplitude *Н_z_* of rod (7) movement by the following dependence:

(6) $$U_{mt}=S_{mt}H_{z},$$

where $S_{mt}$ is the induction transducer sensitivity ($S_{mt}$ = 8.8 mV/mm).

**Figure 6:**
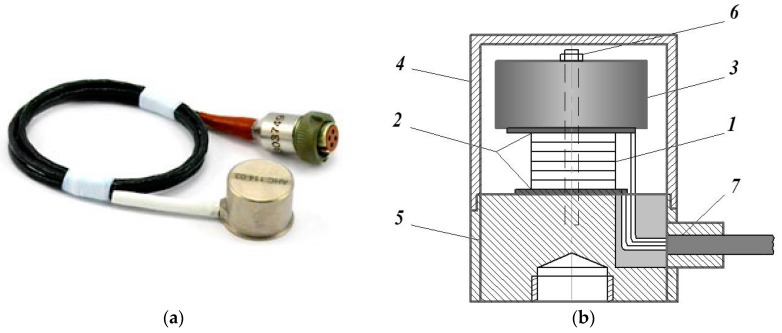
Investigated piezoelectric SE: (**a**) general view; (**b**) construction diagram: 1—PE; 2—insulators; 3—IM; 4—shielding; 5—base; 6—screw; 7—cable.

Investigated SE is shown in Figure 6. It is located on the working table of the mechanical vibration generator GMK-1. The sensing element SE operates on the basis of tension-compression strain.

There is acceleration due to gravity *g_z_* influencing the inertial mass (IM); as a result, IM is displaced by the value *x*:

(7) $$x\equiv f(g_{z}).$$

The movement of IM causes compression or tension of the piezoelectric element (PE) and the appearance of electric charge *Q* (direct piezoelectric effect phenomenon) on its surface, which is directly proportional to *g_z_*. There is a measurement of the PE voltage *U* rather than charge *Q*:

(8) $$U\equiv \frac{Q(g_{z})}{C_{PE}},$$

where $C_{PE}$ is the PE capacity.

Software for the experimental investigation of SE characteristics, i.e., displaying the SE output signal on the PC, is developed on the LabVIEW platform and has the form of a virtual oscilloscope.

## 3. Experimental Investigation of Piezoelectric SE

A piezoelectric accelerometer AHC 114-08 [22], with its natural frequency ω_0_ = 0.1 rad/s, achieved by increasing the total resistance (τ=1CΣRΣ), has been chosen for experimental research. The investigated frequency range was chosen based on the sensor’s natural frequency and typical vibration range experienced by APCs [12].

**Figure 7:**
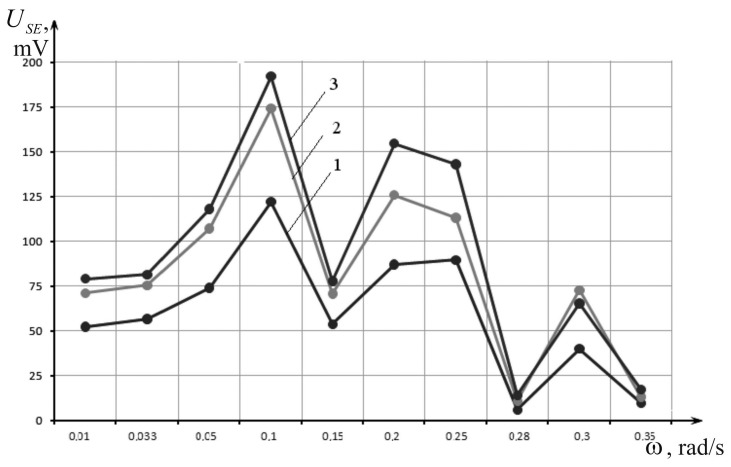
Dependence of SE output voltage on vibration table oscillation frequency at different excitation voltages: 1—$U_{SE}$ = ψ(*ω*) at $U_{gen}$ = 5 V; 2—$U_{SE}$ = ψ(*ω*) at $U_{gen}$ = 7 V; and 3—$U_{SE}$ = ψ(*ω*) at $U_{gen}$ = 8 V.

Dependence of the SE $U_{SE}$ output voltage amplitude on the vibration table oscillation frequency ω for the generator’s voltage amplitude $U_{gen}$ = 5, 7 and 8 V was investigated. The 7 V signal amplitude of the generator was equivalent to the mean amplitude of vibrations experienced by APCs in normal conditions [12]. Lower and higher amplitude was checked for comparison purposes during the sensor’s frequency dependence characterization. The experimental data are shown in the Table 1. Graphs of $U_{SE}$ = *ψ(ω)* for $U_{gen}$ = 5, 7 and 8 V are given in Figure 7.

Table 1 and graphs in Figure 7 show that the maximum output voltage amplitude of the SE investigated takes place when the vibration table oscillation frequency values is ω = 0.1 rad/s for $U_{gen}$ = 5, 7 and 8 V, which equals the frequency of the natural oscillations of the investigated SE (ω = ω_0_ = 0.1 rad/s). This is a case of the so-called “main resonance”. This finding coincides with the findings of analytical studies and PC simulations.

With increasing vibration table oscillation frequency voltage $U_{SE}$ decreases. The experimentally obtained characteristic $U_{SE}$ = ψ(*ω*) is confirmed by the formula $U_{\mathrm{SE}}=\frac{k_{1}k_{2}F_{x}}{\omega S_{x}}$, where $k_{1}=d_{ij}$ is the piezoelectric modulus, and $k_{2}$ is the lithium niobate proportionality coefficient.

**Figure 8:**
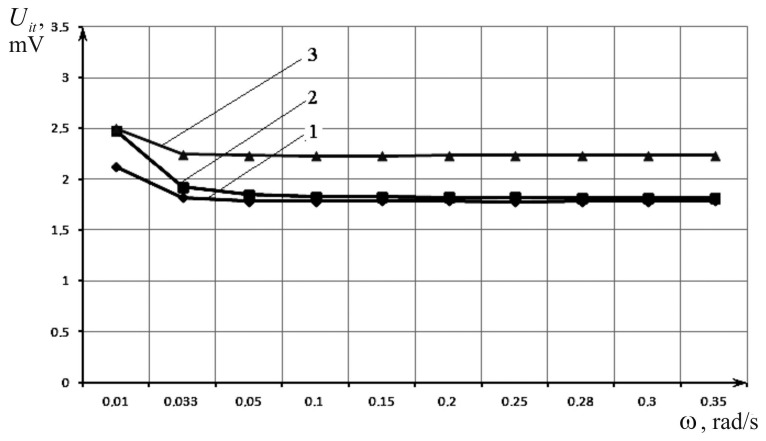
Dependence of the IP output voltage on the vibration table oscillation frequency at different excitation voltages: 1—$U_{it}$ = ψ(*ω*) at $U_{gen}$ = 5 V; 2—$U_{it}$ = ψ(*ω*) at $U_{gen}$ = 7 V; and 3—$U_{it}$ = ψ(*ω*) at $U_{gen}$ = 8 V.

Secondly, research of the dependence of the induction transducer (IT) $U_{it}$ output voltage amplitude on the vibration table oscillation frequency ω for the generator’s voltage amplitude $U_{gen}$ = 5, 7 and 8 V was conducted. The experimental data is shown in Table 2 and dependency graphs of $U_{it}$ = *ψ(ω)* for $U_{gen}$ = 5, 7 and 8 V (Figure 8).

According to the graphs from Figure 8 we conclude that $U_{it}$ does not depend on the vibration table oscillation frequency and is directly proportional to $U_{gen}$. It has also been found that there is deviation from linearity characteristics in the area *ω* ≤ 0.033 rad/s, caused by technological errors of transducer production.

**Figure 9:**
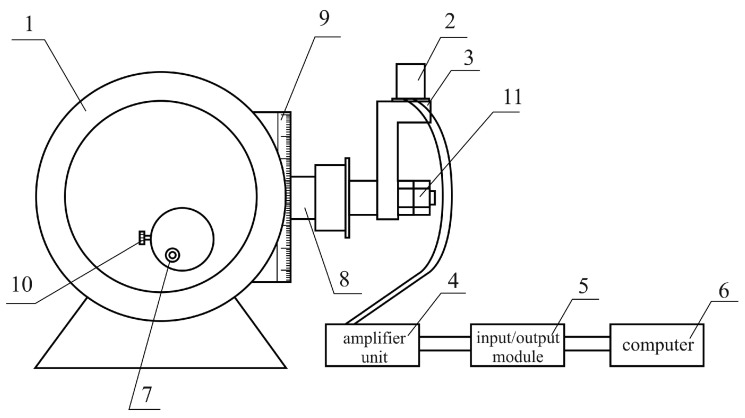
Experimental device for SE calibration 1—optical dividing head; 2—SE; 3—bracket; 4—amplifier unit; 5—input/output module; 6—PC; 7—10: knobs; 8—axle; 9—readout scale; and 11—mounting nuts.

Calibration is a metrological operation that provides measurement instrument (meter or gauge) with a scale or calibration table (curve). For this purpose, we use the device (Figure 9), which consists of an optical dividing head (1), SE (2), mounted on a bracket (3), amplifier unit (4), input/output module (5), and computer (6).

**Figure 10:**
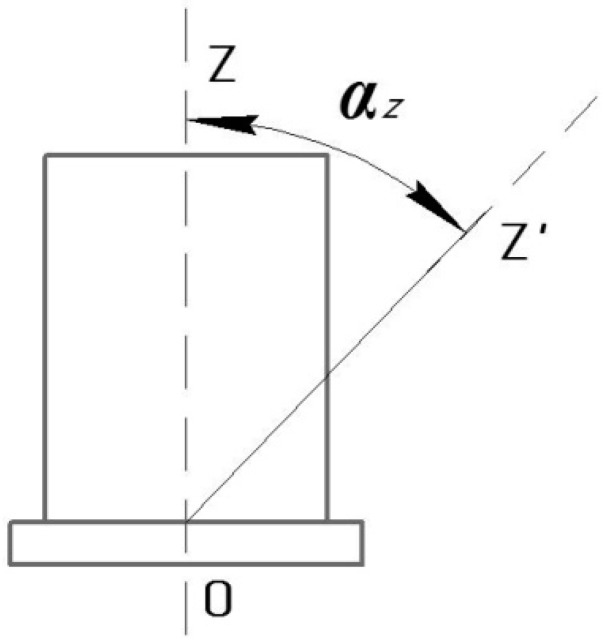
Tilt angle of the SE WSS.

Calibration of the SE takes place when its measurement axis *OZ* is tilted by the optical dividing head at an angle *α_z_* (Figure 10). SE calibration is effected by the twist handle (7) of the optical dividing head (1). This brings the axle (8), the bracket (3), and SE (2) mounted on the bracket into rotation. Rotation angle *α_z_* is adjusted according to the readout scale (9). The output signal of the SE (2) is displayed on the computer (6).

**Figure 11:**
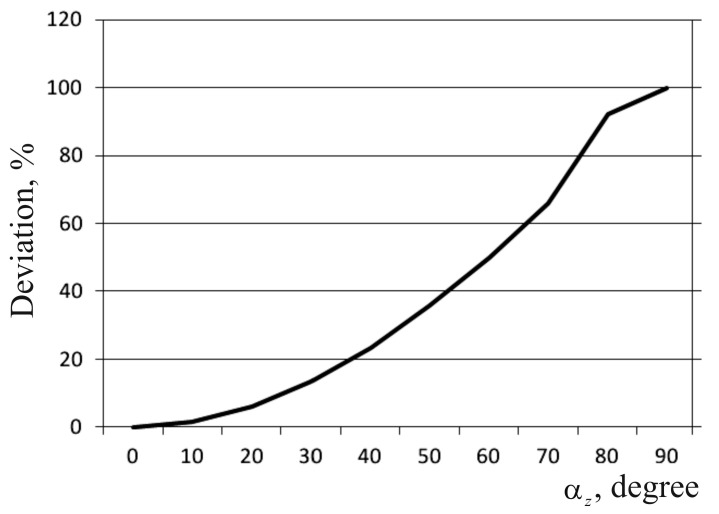
Graph of the dependency of the SE *α_z_* measurement axis deviation on the deviation from the current value of the acceleration due to gravity.

The results $g_{\mathrm{zEXP}}$ of the SE calibration obtained experimentally are displayed in Table 3 and compared with analytical calculations ($g_{\mathrm{zANL}}$ = *g*·cos *α_z_*). Figure 11 shows the constructed graphs of dependency of the SE signal *g_z_* on rotation angle *α_z_*.

As can be seen from the Table 3, the difference between deviations of the measurement axis of the automated WSS SE at angle *α_z_*, calculated analytically and obtained experimentally, is less than 1 mGal. The angle of rotation of its measurement axis relative to the reference vertical directly affects its initial values and the value of its error.

Given that gravimetric measurements are made on the base moving in space, the coincidence of the SE WSS measurement axis with the reference vertical should always be ensured. To put this into practice, it has been proposed to build a stabilization system which provides a level of acceptable error of the SE sensitivity axis stabilization in the vertical position within 0.5–15 arcmin [5].

## 4. Determination of Basic Model Parameters

The device is located at the following GPS coordinates: Longitude: 28.637409°; Latitude: 50.244460°.

According to these coordinates, and the equation [23]:

$$\gamma_{0}=\gamma_{0е}(1+0.0052884\;\sin^{2}\;\phi -0.0000059\;\sin^{2}\;2\phi ),$$

we find a reference value of the acceleration due to gravity $\gamma_{\mathrm{pos}}$:

(9) $$\gamma_{pos}=9.78049(1+0.0052884\;\sin^{2}\;(50.244460)-0.0000059\;\sin^{2}\;(2\times 50.244460))=9.81100376\;{m/s}^{2}.$$

The investigated SE was installed vertically. Tests were conducted on the vibration table shown in Figure 4.

## 5. The Experiment

Data recorded and processed on the computer are presented in Table 4. Data were processed on the PC in about 50 s intervals.

Systematic error $\Delta_{g}$ was calculated for each case:

(10) $$\Delta_{g}=\left|\bar{g_{\mathrm{EXP}}(t)}-\gamma_{pos}\right|,$$

where $\bar{g_{EXP}(t)}$ is the average output SE signal obtained in the experiment during an observation period of 50 s:

(11) $$\bar{g_{EXP}(t)}=\frac{1}{N_{EXP}+1}\sum_{i=0}^{N_{EXP}}\bar{g(t_{i})},$$

where $N_{EXP}$ is the number of measurements during 50 s; $g(t_{i})$ is *i*-th value of the output SE signal.

The absolute error of experimental measurements $\Delta_{g_{EXP}}$ is:

(12) ΔgEXP=σgEXP¯NEXP tp;σgEXP¯=1NEXP∑i=0NEXP[g(ti)¯−gEXP¯]2;tp=qt(p,d),

where $\sigma_{\bar{g_{EXP}}}$ is the standard deviation $\bar{g_{EXP}(t)}$; $t_{p}=qt(p,d)$ is the ratio of Student’s inverse distribution according to confidence probability p, and the number of degrees of freedom.

It has been found from the data summarized in Table 4 that:

- the output SE signal coincides with the reference value of the acceleration due to gravity $\Delta_{g}$ = 0.00006 mGal at the zero setting of the vibration table;
- the SE provides measurement accuracy of $\Delta_{g_{EXP}}$ = 1 mGal for table translational vibration up to 10 rad/s.

According to [6,12] and the modelling results, a resonant mode can occur at frequencies: ω_0_ = 0.033; 0.05; 0.1; 0.2; and 0.3 rad/s.

The spectrum of perturbing translational vibration accelerations in AV has a maximum at a frequency of 1640 rad/s. Therefore, the amplitudes of perturbing translational vibration accelerations are smaller at lower resonant frequencies. Estimation methods of experimental results were not changed. The results are shown in Table 5.

According to Table 5 we conclude that SE provides an accuracy of $\Delta_{g_{EXP}}$ = 1 mGal even in the most unfavourable resonant modes.

It has been established that experimental results are consistent with the results of digital modelling.

## 6. Determination of Metrological Characteristics of Piezoelectric SE

In the absence of linear and angular vibrations, the SE can function as a gravimeter sensor. Theoretical and experimental errors of the SE in the absence of disturbances is 0.00006 mGal = 0.00006 × 10^−5^ m/s^2^.

Thus, the static characteristic of the piezoelectric SE as a gravimetric accelerometer is:

(13) $${\bar{g}}_{SE}=\bar{g_{EXP}}\mp 6\times 10^{-5}\mathrm{mGal}.$$

If there are dynamic disturbances, SE operates as WSS accelerometer with the accuracy of:

(14) $${\bar{g}}_{{\mathrm{SE}}_{\mathrm{WSS}}}=\bar{g_{EXP}}\mp \;1\mathrm{mGal}.$$

The relative error of the SE WSS is:

(15) $$\delta_{g}=\frac{\Delta_{g}}{g_{EXP}}\times 100\%=\frac{1}{981100.37556}\times 100\%=1.019\times 10^{-4}\%.$$

The SE in the WSS has a real-time response, and is limited only by the capabilities of modern computers. Therefore, its speed is high enough.

Operational terms of SE WSS:

- Ambient temperature: −20 to +50 °C;
- Atmospheric pressure: 90,000—110,000 Pa;
- Relative humidity of 50% ± 25%.

The accuracy class of the SE WSS, i.e., the absolute error of the SE under quasi-static laboratory conditions, is $\Delta_{g}$ = 0.00006 mGal, whereas on mobile objects the absolute error is 1 mGal, which meets the highest accuracy class.

## 7. Conclusions

As a result of experimental investigations we have obtained dependencies of amplitudes of the SE output voltage and the induction transducer on the vibration table oscillation frequency. It has been established that the maximum amplitude of the output voltage SE $U_{SE}$ takes place when values of the vibration table oscillation frequency are equal to the values of the SE natural oscillation frequency. This is a case of the so-called “main resonance”. Additionally, voltage $U_{SE}$ decreases with increasing vibration table oscillation frequency.

We have investigated the calibration characteristics of the SE WSS and found that the rotation angle of the SE WSS measurement axis relative to the reference vertical directly impacts on its output values and the value of its error.

SE error has been determined experimentally in the laboratory. It is 0.00006 mGal = 6 × 10^−10^ m/s^2^.

The experiment has shown that the SE provides a measurement accuracy of 1 mGal = 10^−5^ m/s^2^ in the most adverse resonant conditions, *ω* = *ω*_0_ = 0.1 rad/s, *ω* = 2*ω*_0_, *ω* = 3*ω*_0_, *ω* = *ω*_0_/2, *ω* = *ω*_0_/3.

It has been found that systematic error of the SE is at its maximum at ω = 3ω_0_ = 0.3 rad/s and does not affect measurement accuracy.

The main sources of the sensor’s error are the temperature coefficient of piezoelectric modulus for given material, mass and dimension variations between individual sensors, and supply voltage stabilization errors. It is thoroughly analysed in [24]. Another source of error are the hysteresis factors of piezoelectric elements, modelling, and measurement of which are described in [25,26]. The identification and compensation of hysteresis effects should be included in future developments of the sensor, if greater accuracy is required.

The new sensor is implemented in the SVU-500 WSS, and produced by the G. Petrovsky Kyiv Automatics Plant for a modernised version of the BMP-2 APC. Newly-produced units are currently (late-2016) being investigated in military trials. Preliminary results indicate an order of magnitude improvement of the modernised system accuracy.

## Figures and Tables

**Table 1:** The experimental data.

*ω* (Rad/s)		0.01	0.033	0.05	0.1	0.15	0.20	0.25	0.28	0.30	0.35
$U_{SE}$ (mV)	At $U_{gen}$ = 5 V	52.4	56.7	74	121.9	53.8	87	89.6	6.1	40.0	9.8
	At $U_{gen}$ = 7 V	71	75.5	107.1	174.1	70.7	125.8	113	10.8	72.6	13.2
	At $U_{gen}$ = 8 V	79	81.3	118	192	77.8	154.6	143	14	65.2	17

**Table 2:** Dependence of the IT output voltage on the vibration table oscillation frequency at different excitation voltages.

*ω* (Rad/s)		0.01	0.033	0.05	0.1	0.15	0.20	0.25	0.28	0.30	0.35
$U_{it}$ (V)	$U_{gen}$ = 5 V	2.120	1.820	1.780	1.782	1.786	1.785	1.779	1.784	1.782	1.787
	$U_{gen}$ = 7 V	2.480	1.920	1.850	1.830	1.829	1.822	1.821	1.818	1.819	1.813
	$U_{gen}$ = 8 V	2.50	2.250	2.240	2.230	2.230	2.240	2.240	2.240	2.240	2.240

**Table 3:** Calibration table of the SE WSS.

No.	*α_z_* (°)	$g_{\mathrm{zEXP}}$ (mGal)	$g_{\mathrm{zANL}}$ (mGal)	Module of Deviation of Experimental Data from Theoretical Data (mGal)	Deviation from the Current Value (%)
1	2	3	4	5	6
1	0	981,100.375	981,100.376	0.001	0
2	10	966,195.234	966,195.257	0.023	1.52
3	20	921,932.665	921,932.784	0.119	6.03
4	30	849,658.072	849,657.849	0.223	13.39
5	40	751,566.893	751,566.491	0.402	23.40
6	50	630,639.662	630,639.161	0.501	35.72
7	60	490,549.470	490,550.188	0.718	50.01
8	70	335,556.981	335,556.091	0.890	65.79
9	80	17,365.725	17,364.818	0.907	98.23
10	90	0	0	0	100

**Table 4:** SE errors caused by vibrations of the base at *p* = 0.90.

*ω* (Rad/s)	$\bar{g_{EXP}(t)}$ (mGal)	$\Delta_{g_{EXP}}$ (mGal)	$\Delta_{g}$ (mGal)
0	981,100.3761	0.001136	0.00006001
0.5	981,103.2946	0.006184	2.91861022
1.0	981,103.4298	0.048067	3.05381611
5.0	981,105.7721	0.581020	5.39611120
10.0	981,108.9362	0.851001	8.89863610
30.0	981,113.4471	2.764100	13.0710563

**Table 5:** SE errors caused by resonant modes.

*ω* (Rad/s)	$\bar{g_{EXP}(t)}$ (mGal)	$\Delta_{g_{EXP}}$ (mGal)	$\Delta_{g}$ (mGal)
0	981,100.3761	0.001136	0.000060
0.033	981,100.5046	0.191160	0.128636
0.05	981,100.5798	0.378130	0.203863
0.1	981,101.1799	0.962309	0.803863
0.2	981,101.5961	0.411891	1.220125
0.3	981,102.4886	0.384961	2.112581
